# Rapid Emergence of Novel GII.4 Sub-Lineages Noroviruses Associated with Outbreaks in Huzhou, China, 2008–2012

**DOI:** 10.1371/journal.pone.0082627

**Published:** 2013-12-04

**Authors:** Lei Ji, Xiaofang Wu, Wenting Yao, Liping Chen, Deshun Xu, Yuehua Shen, Jiayu Shen, Jiankang Han

**Affiliations:** Huzhou Center for Disease Control and Prevention, Huzhou, Zhejiang Province, China; National Institute for Viral Disease Control and Prevention, CDC, China

## Abstract

Infection caused by noroviruses (NoVs) is one of the most important causes of acute gastroenteritis in humans worldwide. To gain insight into the epidemiology of and genetic variation in NoV strains, stool samples collected from 18 outbreaks of acute gastroenteritis in Huzhou, China, between January 2008 and December 2012 were analyzed. Samples were tested for NoVs by real-time RT-PCR. Partial sequences of the RNA- dependent RNA polymerase (RdRp) and capsid gene of the positive samples were amplified by RT-PCR, and the PCR products were sequenced and used for phylogenetic analysis. NoVs were found to be responsible of 88.8% of all nonbacterial acute gastroenteritis outbreaks in Huzhou over the last 5 years. Genogroup II outbreaks largely predominated and represented 93% of all outbreaks. A variety of genotypes were found among genogroups I and II, including GI.4, GI.8, GII.4, and GII.b. Moreover, phylogenetic analyses identified two recombinant genotypes (polymerase/capsid): GI.2/GI.6 and GII.e/GII.4 2012 Sydney. GII.4 was predominant and involved in 8/10 typed outbreaks. During the study period, GII.4 NoV variants 2006b, New Orleans 2009, and Sydney 2012 were identified. This is the first report of the detection of GII.4 New Orleans 2009 variant, GII.e/GII.4 Sydney 2012 recombinant in outbreaks of acute gastroenteritis in China.

## Introduction

Noroviruses (NoVs) are the major cause of outbreaks and sporadic cases of nonbacterial acute gastroenteritis in all age groups in both developing and developed countries. Transmission can occur through direct contact with people shedding the virus, contaminated food, sewage-contaminated water, contaminated aerosols, and environmental contamination. NoVs are highly infectious due to the low infectious dose, high stability in the environment, and short-term host immunity. Most NoV outbreaks occur in semi-closed communities, such as the military, hospitals, schools, and cruise ships, which favor person-to-person spread and are difficult to control [1]. 

NoVs are single-stranded, positive-sense RNA viruses that belong to the *Caliciviridae* family and possess a polyadenylated RNA genome of 7.3–7.7 kb containing three overlapping open reading frames (ORFs) [2]. ORF1 encodes a polyprotein that is processed posttranslationally into several nonstructural proteins, including the RNA-dependent RNA polymerase (RdRp). ORF2 encodes the major structural protein (capsid protein, VP1) and ORF3 encodes a minor structural protein (VP2) [3]. NoVs are genetically highly diverse and can be grouped into five genogroups (GI through GV), which are further divided into at least 34 genotypes based on genetic differences in the capsid gene [4]. Human disease is primarily caused by GI and GII noroviruses, with most outbreaks caused by GII.4 strains [5,6]. The high degree of genetic diversity of NoVs can be attributed to both point mutations during genome replication and RNA recombination between co-circulating strains. During the past decade, new subtypes or variants of GII.4 strains have emerged every 2–3 years and usually became the dominant strains in the new seasons [7]. Recombination of NoV genomes is believed to occur in nature at high frequency. The majority of NoV recombinants have breakpoints located either within or close to the ORF1-ORF2 junction, which causes more genetic divergence of NoVs [8,9]. Since it was first reported [10], many recombinant NoVs from different genotypes and genogroups have been described.

The absence of a cell culture system or small animal model for human NoVs has led to the development of molecular tools to detect and characterize of NoVs. Surveillance has indicated that NoVs are the most important nonbacterial cause of sporadic gastroenteritis in both children and adults in China [11 12 13–14]. However, the molecular epidemiology of NoV infections in nonbacterial acute gastroenteritis outbreaks in China has not been well studied. To gain insight into the epidemiological patterns of NoV outbreaks and to determine the genetic variation in NoV strains, we tested stool samples collected during acute gastroenteritis outbreaks, between January 2008 and December 2012 in Huzhou city, Zhejiang, located in northern Zhejiang province, China.

## Materials and Methods

### Ethics statement

Institutional review board approval was not requested, as this study was part of a routine laboratory-based outbreak investigation. The only human materials used were stool samples collected from patients in gastroenteritis outbreaks for public health purposes. All participants were voluntarily cooperative in providing specimens and questionnaire information during case investigation. Patient consent was not required as they were tested as per routine outbreak laboratory investigations and patient demographic information was not included in the analysis.

### Samples and outbreaks

Fecal specimens obtained from nonbacterial outbreaks of acute gastroenteritis that occurred in the Huzhou area from January 2008 through December 2012 were collected. An outbreak of gastroenteritis was defined as two or more cases of acute gastroenteritis occurring in a given setting within a period of 2 weeks. All outbreaks of acute gastroenteritis should be reported to the local health departments. The Centers for Disease Control and Prevention is responsible for outbreak investigations, epidemiological consultation, and laboratory support. All samples had already been shown to be negative for intestinal pathogenic bacteria. Specimens were stored at –70°C. 

### RNA extraction

Viral RNA was extracted from 10% stool suspension in phosphate-buffered saline (pH 7.4) with a QIAamp viral RNA mini kit (Qiagen, Hilden, Germany) according to the manufacturer’s instructions. The RNA extracts were used directly for reverse transcription (RT)-PCR or stored at –70°C.

### Detection of NoV by RT-qPCR

Real-time PCR (qPCR) was performed using primers and probes to detect the majority of the human GI and GII NoV strains as described previously (Table S1) [15]. RT-qPCR was carried out using a One Step PrimeScript® RT-PCR Kit (Perfect Real Time) (TaKaRa, Dalian, China). Briefly, the 25 µL reaction mixture consisted of 1×RT-PCR buffer, 2.5 U of EX Taq HS polymerase, 0.5µL RT Enzyme Mix II, 400 nM of each primer (JJV1F, JJV1R, JJV2F, and COG2R), 100 nM of each probe (JJV1P and RING2-TP), and 5µL of viral RNA. RT was performed at 42°C for 30 min, followed by 95°C for 5 min and 40 cycles of qPCR at 95°C for 5 s and 55°C for 35 s. 

### Genomic amplification for genotyping

For all samples that tested positive for NoV by RT-qPCR, viral RNA was extracted from 140 μL clarified fecal suspension using a QIAamp viral RNA mini kit. RNA extracts were stored at –70°C. For genotyping, published primers were used for amplification and sequencing of the 3' of the polymerase gene (region A in ORF1) and the 5' end of the capsid gene (region C in ORF2) (Table S2) [16,17]. For potential recombinant genomes of NoVs, sequences covering the overlap between ORF1 and ORF2 were amplified by RT-PCR using primers JV12 and G1SKR/G2SKR when phylogenetic analyses indicated incongruent clustering for partial sequences of the polymerase and capsid genes in the same samples. RT-PCR was carried out using a One Step RNA PCR Kit (TaKaRa). RT-PCR conditions were as follows: RT at 50°C for 30 min and denaturation at 95°C for 2 min, followed by 35 cycles of 30 s at 94°C, 30 s at 48°C, and 1 min at 72°C. A final elongation step was performed for 10 min at 72°C. After amplification, the PCR products were visualized by electrophoresis. 

### Sequencing

The PCR products were purified using a QIAquick PCR purification kit (Qiagen, Leusden, The Netherlands) according to the manufacturer’s instructions. Direct sequencing of PCR products was carried out by TaKaRa Biotechnology (Dalian).

### Molecular typing and phylogenetic analysis

Genotypes of NoVs were preliminarily assigned using BLAST and then confirmed using a Web-based NoV genotyping tool (http://www.rivm.nl/mpf/norovirus/typingtool) [18]. Phylogenetic and molecular evolutionary analyses were conducted using the MEGA program, version 5 [19]. A phylogenetic tree was generated using the neighbor-joining algorithm with the Kimura two-parameter model and supported statistically by bootstrapping with 1000 replicates. Recombination analysis was conducted using the SimPlot program (v 3.5) [20].

### Nucleotide sequence accession numbers

The GenBank accession numbers for sequences obtained for the partial capsid gene and partial polymerase in this study follow: HQ680712-HQ680721, HQ840433-HQ840434, JX644025-JX644039, KC473544-KC473548, KF055278-KF055286, KF048034-KF048039.

## Results

### Characteristics of nonbacterial acute gastroenteritis outbreaks in Huzhou from 2008 to 2012

From January 2008 to December 2012, 155 stool specimens from 18 nonbacterial acute gastroenteritis outbreaks in the Huzhou area were examined by RT-qPCR that detects NoVs in a genogroup-specific manner. A total of 77 of the 155 (49.7%) specimens were positive for NoVs and samples from 2 of the 18 outbreaks tested negative for NoV. NoVs were involved in 16 (88.8%) nonbacterial acute gastroenteritis outbreaks (Table 1), including one outbreak in 2008, three outbreaks in 2009, five outbreaks in 2010, four outbreaks in 2011, and three outbreaks in 2012. NoV-associated gastroenteritis outbreaks occurred throughout the year. Although outbreaks occurred in a variety of settings, more than half (68%) occurred in schools. From 2008 to 2012, GII NoV outbreaks predominated and represented 93% (15/16) of the NoVs in either single infections or in co-infection with GI NoVs. GI single infections and GI-GII co-infections represented only 6% (1/16) and 18% (3/16) of the outbreaks, respectively.

**Table 1:** Epidemiological data of norovirus gastroenteritis outbreaks and genogroup/genotype information of norovirus-positive samples.

**Year**	**Time (Year. Month)**	**Setting**	**Positive/tested samples**	**Genogroup (RT-qPCR)**	**Strain**	**Genotype (RT-PCR)**	
						Region A	Region C
2008	2008.4	Middle school	13/18	GI GII	N2, N3, N4, N5, N8	GII.4 2006b	GII.4 2006b
					N11, N12	GI.2	GI.6
					N1, N10	GI.8	GI.8
2009	2009.2	Middle school	9/5	GI GII	N22	GI.8	GI.8
					N23	GII.b	–
	2009.8	unknown	5/5	GII	N13	GII.4 2006b	GII.4 2006b
	2009.10	Middle school	2/11	GII	–	–	–
2010	2010.4	Middle school	3/19	GII	–	–	–
	2010.5	Kinder garden	5/5	GII	–	–	–
	2010.11	Elementary school	4/18	GII	–	–	–
	2010.11	University	5/10	GI GII	–	–	–
	2010.12	Village	3/3	GII	N76, N78	GII.4 New Orleans 2009	GII.4 New Orleans 2009
2011	2011.1	Elementary school	8/8	GII	N82	GII.4 2006b	GII.4 2006b
	2011.2	Hospital	6/8	GII	N91, N93, N94, N95	GII.4 New Orleans 2009	GII.4 New Orleans 2009
	2011.2	Elementary school	3/4	GII	–	–	–
	2011.10	Village	2/2	GII	N100, N101	GII.4 New Orleans 2009	GII.4 New Orleans 2009
2012	2012.5	Elementary school	1/9	GI	N111	GI.4	GI.4
	2012.11	Village	3/3	GII	N121, N122	GII.e	GII.4 Sydney 2012
	2012.11	Middle school	5/7	GII	N128	GII.e	GII.4 Sydney 2012

A: partial polymerase gene; C: partial capsid gene.

### Genotyping

Classification of NoVs into genogroups GI and GII was possible for all samples that tested positive by RT-qPCR but more accurate genotyping was only possible for just over half of the NoV outbreaks, and no genotyping information could be obtained for six outbreaks (37.5%) (Table 1). Phylogenetic analyses were performed with 25 NoV-positive samples from 10 outbreaks for the partial polymerase (*n* = 25) and/or capsid gene (*n* = 24) sequences. Phylogenetic trees were drawn for both the partial polymerase and capsid gene sequences; genotyping results for the amplified region of each sample are shown in Table 1.

**Figure 1:**
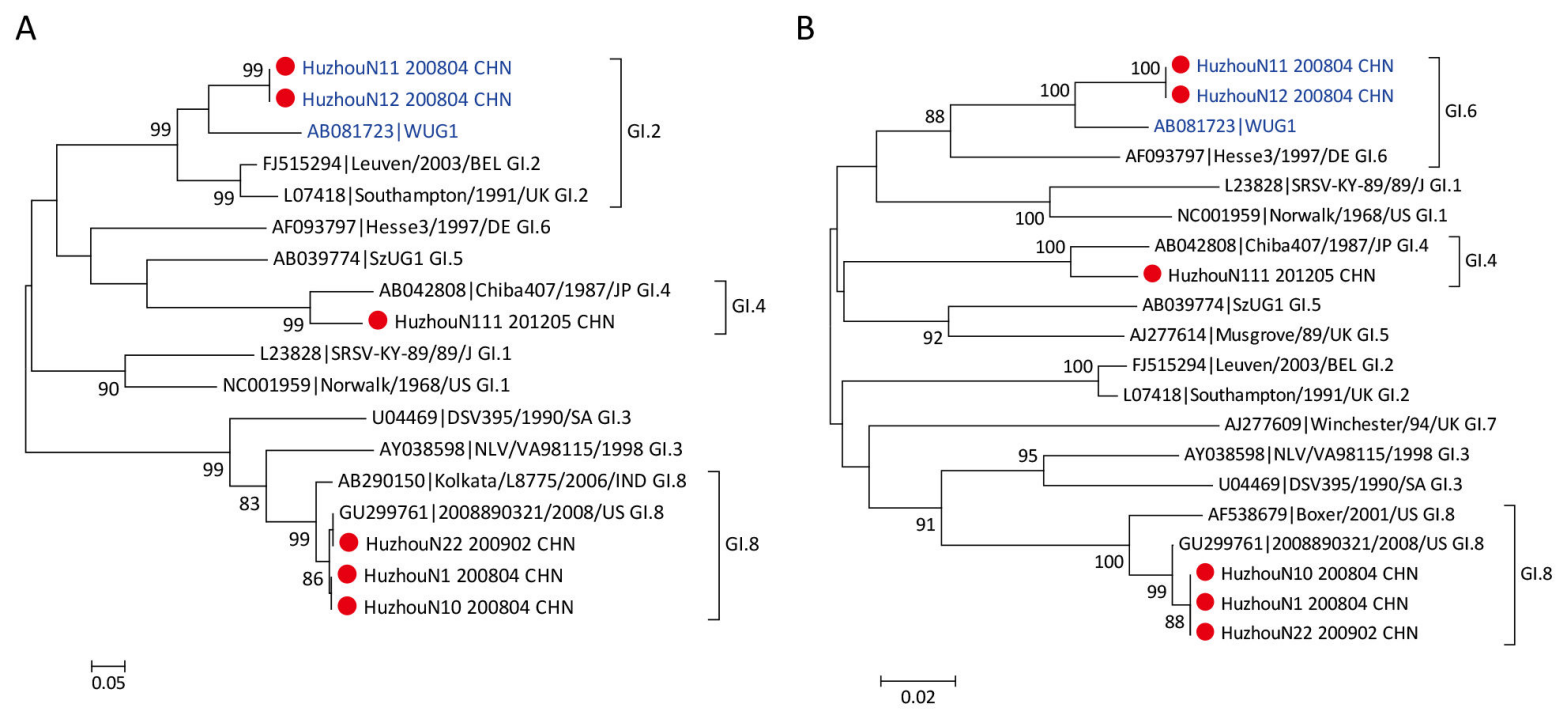
Phylogenetic analyses of GI noroviruses detected in Huzhou. Phylogenetic analyses of the partial RdRp region (A) and capsid region (B) of GI noroviruses. Norovirus strains detected from Huzhou are denoted by red circles. The GI.2/GI.6 recombinants identified in this study and reference strains are highlighted in blue. The reference strain names are formatted as GenBank accession number|strain name. The tree was constructed using the neighbor-joining method and bootstrap values ≥ 80% are shown.

Phylogenetic analysis clustered GI NoVs from three outbreaks into three different genotypes: GI.4, GI.8, and recombinant GI.2/GI.6 (polymerase/capsid) (Figure 1A and 1B). One single GI NoV outbreak was successfully genotyped and caused by GI.4, whereas GI.2/GI.6 and GI.8 strains were amplified from samples originating from two outbreaks involving GI and GII co-infection that occurred in the same school. The GI.8 strains (N1, N10, and N22) originating from the two outbreaks in the same school were closely related, with 97.9–99.5% similarity between the partial polymerase and capsid gene sequences.

**Figure 2:**
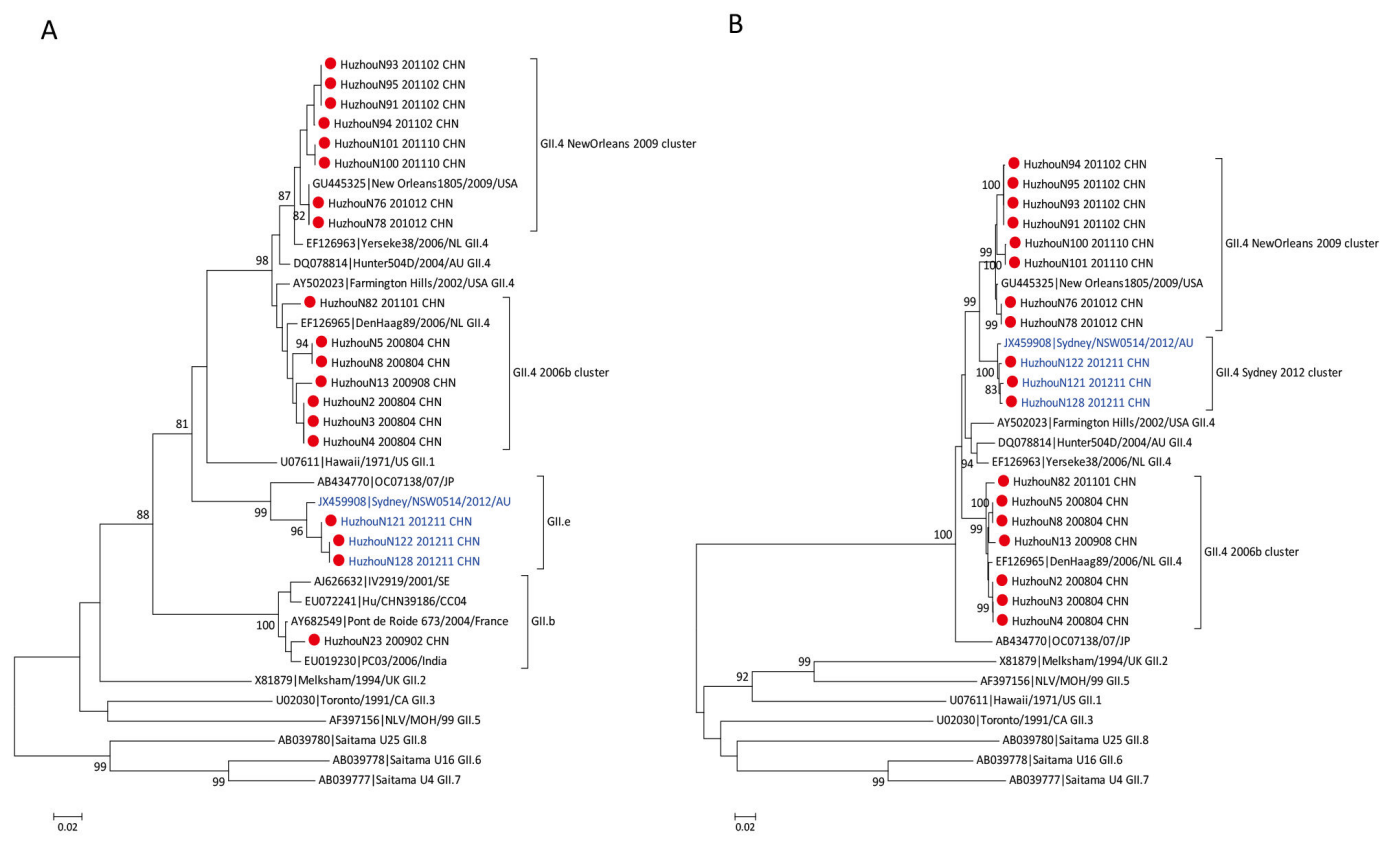
Phylogenetic analyses of GII noroviruses detected in Huzhou. Phylogenetic analyses of the partial RdRp region (A) and capsid region (B) of GII noroviruses. Norovirus strains detected from Huzhou are denoted by red circles. The GII.e/GII.4 recombinants identified in this study and reference strains are highlighted in blue. Reference strain names are formatted as GenBank accession number|strain name. The tree was constructed using the neighbor-joining method and bootstrap values ≥ 80% are shown.

Phylogenetic analysis clustered GII NoVs into three different genotypes: GII.4, GII.b, and recombinant GII.e/GII.4 (polymerase/capsid). All of the sequenced GII NoVs belonged to the GII.4 genotype in polymerase and/or capsid genes with the exception of strain N23 (Figure 2A and 2B), which had a GII.b polymerase gene. Moreover, amplification of its capsid protein region failed. NoV recombinants with GII.b polymerase and GII.1, GII.2, GII.3, or GII.4 capsid caused hundreds of NoV outbreaks throughout Europe between 2000 and 2001 [21 22–23]. They have also been detected subsequently in many Asian countries, including Japan, India, and China [24 25 26–27]. The partial polymerase sequence of strain N23 showed a high level of identity (96.0–98.8%) to the GII.b reference strains isolated from these Asian and European countries.

Phylogenetic clustering with a GII.4 variant reference set from the NoroNet European genotyping tool allowed characterization of GII.4 NoV circulating during the study period. GII.4 NoV sequences grouped into three different clusters for the polymerase and capsid genes, respectively (Figure 2A and 2B). In addition to the pandemic variants GII.4 2006b, GII.4 sequences clustered with GII.4 New Orleans 2009 variant and the newly reported GII.4 Sydney 2012 variant (for the capsid gene only). In the Huzhou area, the 2006b variants were detected until January 2011 and in co-circulation with GII.4 New Orleans 2009 variants in 2011; subsequently, GII.4 Sydney 2012 was the only GII.4 variant detected in outbreaks in 2012. 

### Identification of NoV recombinants

**Figure 3:**
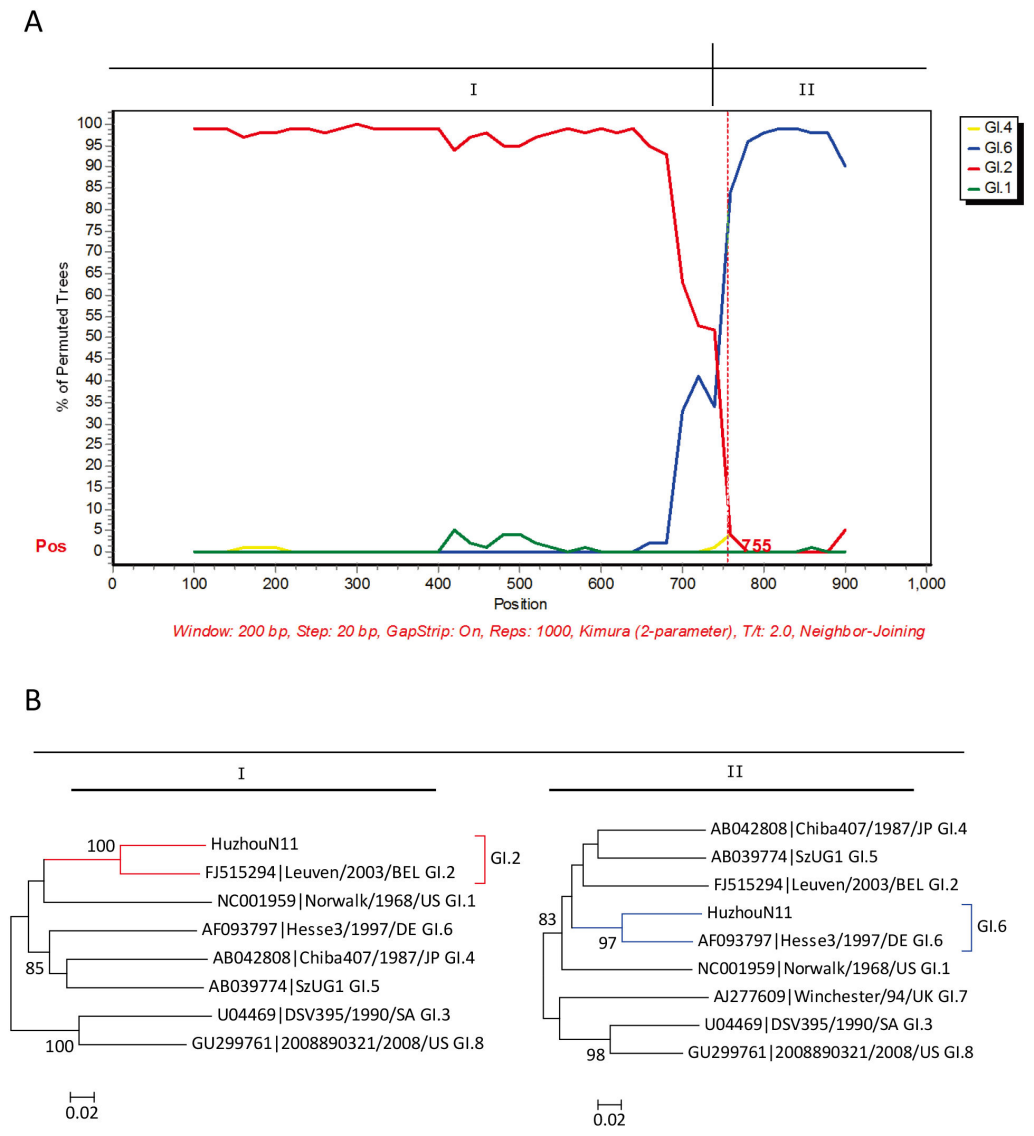
Bootscanning plot of sequences covering the ORF1/2 overlap of GI. 2/GI.6 recombinant HuzhouN11. (A) Bootscanning plot of HuzhouN11 using GI.1 (Norwalk/1968/US), GI.2 (Leuven/2003/BEL), GI.4 (Chiba407/1987/JP), and GI.6 (Hesse3/1997/DE) as subtype references. Dashed vertical lines indicate the start of the capsid gene. (B) Phylogenetic analyses of segment I (3'-ORF1) and segment II (5'-ORF2) defined by bootscanning. The phylogenetic trees were constructed with MEGA 5.0 using the neighbor-joining method. Segment I of HuzhouN11 clustered with the GI.2 subtype reference and segment II of HuzhouN11 clustered with the GI.6 subtype reference.

**Figure 4:**
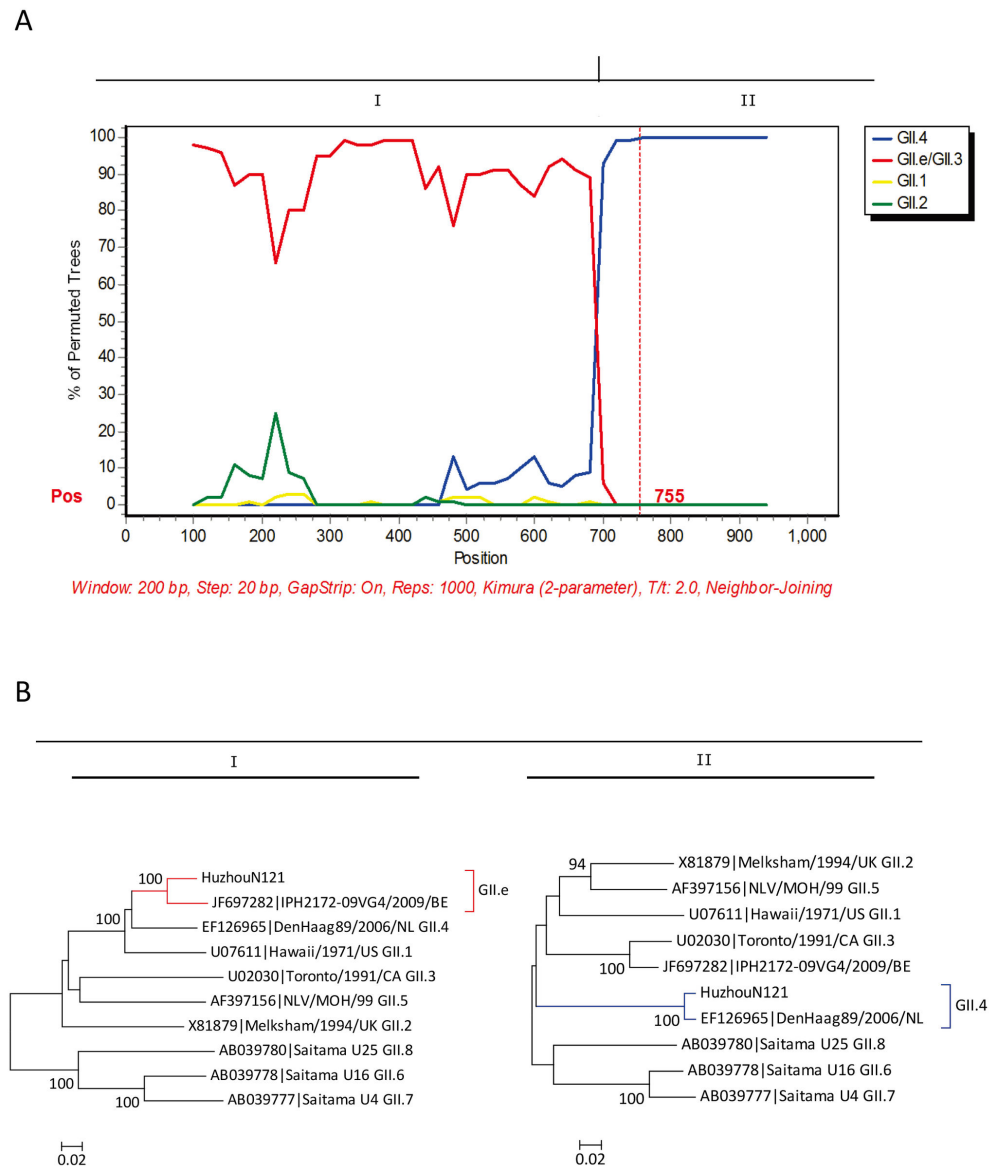
Bootscanning plot of sequences covering the ORF1/2 overlap of GII. e/GII.4 recombinant HuzhouN121. (A) Bootscanning plot of HuzhouN121 using GII.1 (Hawaii/1971/US), GII.2 (Melksham/1994/UK), GII.4 (DenHaag89/2006/NL), and GII.e/GII.3 (IPH2172–09VG4/2009/BE) as subtype references. Dashed vertical lines indicate the start of the capsid gene. (B) Phylogenetic analyses of segment I (3'-ORF1) and segment II (5'-ORF2) deﬁned by bootscanning. The phylogenetic trees were constructed with MEGA 5.0 using the neighbor-joining method. Segment I of HuzhouN121 clustered with the GII.e subtype reference and segment II of HuzhouN121 clustered with the GII.4 subtype reference.

The identification of inconsistent genotype clustering for the partial polymerase and capsid gene sequences for NoV strains allowed the detection of two types of recombinants involved in outbreaks: GI.2/GI.6 and GII.e/GII.4 Sydney 2012. The GI.2/GI.6 recombinant strains in this study shared 88.3% and 94.0% nucleotide sequence identity with the GI.2/GI.6 prototype WUGI/01/JP in the partial polymerase and capsid gene, respectively. Similar to the GII.4 Sydney 2012 reference strain NSW0514/2012/AU, the GII.4 2012 variant identified in this study had the GII.e polymerase gene for which no associated capsid genes have been described, and shared 82.0–86.3% nucleotide identity with the reference strain OC07138/07/JP (GII.e/GII.4 recombinant) in the polymerase region. For representative strains with potential recombinant genotypes of GI.2/GI.6 (HuzhouN11) and GII.e/GII.4 Sydney 2012 (HuzhouN121), sequences covering the ORF1-ORF2 junction were obtained to study recombination breakpoints using SimPlot software. Bootscanning plot analyses confirmed that all recombination points were located close to the ORF1-ORF2 junction (Figures 3A and 4A), which corresponds to the previously proposed hotspot for NoV recombination [8,9]. At the breakpoint position, Huzhou N11 and HuzhouN121 were divided into two segments (I and II), respectively. To further confirm the NoV recombinants identified by SimPlot, phylogenetic analyses of the two segments (I and II) defined by bootscanning were conducted with MEGA 5.0. Segment I of HuzhouN11 was clustered with the GI.2 subtype reference strain, while segment II of HuzhouN11 was clustered with the GI.6 subtype reference strain (Figure 3B). Segment I of HuzhouN121 was clustered with the GII.e subtype reference strain, while segment II of HuzhouN121 was clustered with the GII.4 subtype reference strain (Figure 4B).

## Discussion

A systematic investigation of the molecular epidemiology of NoVs detected in nonbacterial acute gastroenteritis outbreaks was conducted in the Huzhou area from January 2008 to December 2012. NoV was detected in two or more samples from 88.8% of the outbreaks. The outbreaks occurred throughout the year, and more than half occurred in schools. Genotyping analyses identified at least four genotypes (GI.4, GI.8, GII.4, GII.b) and two recombinant genotypes (polymerase/capsid, GI.2/GI.6 and GII.e/GII.4 Sydney 2012). GII.4 subtyping identified the circulation of three different variants in the Huzhou area since 2008, i.e., 2006b, New Orleans 2009, and Sydney 2012.

 Since 1995, most NoV outbreaks worldwide have been caused by variants of NoVs belonging to the GII.4 lineage. Compared to other NoVs, those belonging to this lineage undergo influenza virus-like antigenic drift (genetic drift as a major evolutionary force), which results in the selection of new pandemic variants [28,29]. In the last decade, new GII.4 pandemic variants have emerged every 2–3 years and usually became the dominant strains in the new seasons associated with an increase in disease activity [7,30]. For example, this was the case in 2002 (Farmington Hills variant), 2004 (Hunter variant), 2007 (2006b variant), and 2010 (New Orleans 2009 variant). Until 2009, GII.4 2006b variants were still the predominate strains in many countries, including China [11,31]. Since 2010, the newly emerged GII.4 New Orleans 2009 variant seems to have displaced the 2006b sublineage and to have become dominant worldwide [32 33–34].

We also observed similar patterns of circulating GII.4 variants. In the Huzhou area, GII.4 2006b variants were detected until January 2011 and constituted the only GII.4 variant circulating in 2008 and 2009. Since the first detection of the GII.4 New Orleans 2009 variant in December 2010, they have been in co-circulation with GII.4 2006b; from 2010 to 2011, they accounted for three of four (75%) typed outbreaks. 

In March 2012, a new GII.4 NoV strain was identified in Australia, which was named GII.4 Sydney 2012. In late 2012, various countries around the globe reported higher incidences of NoV outbreaks or illness [29,35,36]. The molecular data uploaded to the international molecular surveillance database NoroNet indicate that all of these increases were associated with the emergence of GII.4 Sydney 2012 variants [35]. Beginning in July 2012, a community-wide increase in the number of GII.4 2012-associated acute gastroenteritis cases was observed in Hong Kong, China [37]. In the Huzhou area, highly similar GII.4 Sydney 2012 variants were also identified in November 2012, which caused two outbreaks in November and became the only GII.4 variant detected in outbreaks in 2012, suggesting its widespread dissemination, including China. 

Recombination between NoV genomes has occurred frequently as another important feature of the evolution of NoVs. We also detected two recombinant NoVs in this study, GII.e/GII.4 Sydney 2012 and GI.2/GI.6. As described previously for GII.b recombinants, several different capsid sequences can be associated with the GII.e polymerase genes [38]. It seems that the association between these polymerases and distinct capsid gene sequences could provide some selective advantages over monophylogenetic strains. During 2008–2009, GII.e polymerases in association with GII.4 2007 or GII.3 capsids were the second most prevalent genotype after GII.4 in Belgium [32]. Since late 2012, the emergence of GII.e/GII.4 Sydney 2012 has caused acute gastroenteritis outbreaks or illnesses in multiple countries. The identification of GII.e/GII.4 2012 recombinants in many countries in 2012 suggests that they emerged after undergoing a recent recombination event. Another recombinant identified in this study was GI.2/GI.6, originating from two outbreaks in which GI and GII were co-infections. The GI.2/GI.6 recombinant was a common GI recombination type and has been detected in many countries [9,39]. It has also been detected in sporadic cases of acute gastroenteritis in adults in Beijing, China [11].

In conclusion, our data indicate that NoVs are the most important cause of nonbacterial acute gastroenteritis outbreaks in the Huzhou area. Although highly diverse, NoVs circulating in Huzhou over the past 5 years were predominantly GII.4 sublineages, including GII.4 variants 2006b, New Orleans 2009, and Sydney 2012. To the best of our knowledge, this is the first report regarding the detection of GII.4 New Orleans 2009 variant, GII.e/Sydney 2012 recombinant in outbreaks of acute gastroenteritis in China. The new variant or recombinant strains of NoV originating in other countries can spread into China very quickly, especially the GII.4 variants. As the epidemiology of NoV strains may change rapidly, continuous surveillance focusing on strain variation and dynamic change is necessary for understanding the molecular epidemiology of NoV infections and development of improved control and prevention strategies.

## Supporting Information

Table S1 **Primer and probe oligonucleotides used for real-time quantitative RT-PCR.**
(DOC)

Table S2 **Oligonucleotide primers used for genotyping.**
(DOC)
