# Supplementary material for: Rapid Emergence of Novel GII.4 Sub-Lineages Noroviruses Associated with Outbreaks in Huzhou, China, 2008–2012
Source: PLoS One. 2013 Dec 4;8(12):e82627. doi: 10.1371/journal.pone.0082627 (PMC3853588; doi:10.1371/journal.pone.0082627)
Supplement: Table S1 — Primer and probe oligonucleotides used for real-time quantitative RT-PCR. (DOC) [file pone.0082627.s001.doc]

Table S1 Primer and probe oligonucleotides used for real-time quantitative RT-PCR

| Genogroup | Primer or probe | Polarity | Location^a^ | Sequence (5′-3′) |
| --- | --- | --- | --- | --- |
| GI | JJV1F | + | 5282-5299 | GCCATGTTCCGITGGATG |
|  | JJV1R | - | 5358-5377 | TCCTTAGACGCCATCATCAT |
|  | JJV1P | + | 5319-5341 | FAM-TGTGGACAGGAGATCGCAATCTC-TAMRA |
| GII | JJV2F | + | 5003-5028 | CAAGAGTCAATGTTTAGGTGGATGAG |
|  | COG2R | - | 5080-5100 | TCGACGCCATCTTCATTCACA |
|  | RING2-TP | + | 5048-5067 | FAM-TGGGAGGGCGATCGCAATCT-TAMRA |

^a^Position in the norovirus genomic sequence GI.1 (M87661)
