# Supplementary material for: Rapid Emergence of Novel GII.4 Sub-Lineages Noroviruses Associated with Outbreaks in Huzhou, China, 2008–2012
Source: PLoS One. 2013 Dec 4;8(12):e82627. doi: 10.1371/journal.pone.0082627 (PMC3853588; doi:10.1371/journal.pone.0082627)
Supplement: Table S2 — Oligonucleotide primers used for genotyping. (DOC) [file pone.0082627.s002.doc]

Table S2 Oligonucleotide primers used for genotyping

| Genogroup | Region | Primer | Polarity | Location^a^ | Sequence (5′-3′) |
| --- | --- | --- | --- | --- | --- |
| GI、GII | A | JV12 | + | 4552–4572 | ATACCACTATGATGCAGATTA |
|  |  | JV13 | - | 4858–4878 | TCATCATCACCATAGAAAGAG |
| GI | C | G1SKF | + | 5342-5361 | CTGCCCGAATTYGTAAATGA |
|  |  | G1SKR | - | 5652-5671 | CCAACCCARCCATTRTACA |
| GII | C | G2SKF | + | 5046-5064^b^ | CNTGGGAGGGCGATCGCAA |
|  |  | G2SKR | - | 5367-5389 | CCRCCNGCATRHCCRTTRTACAT |

^a^Position in the norovirus genomic sequence GI.1 (M87661)

^b^ Position in the norovirus genomic sequence GII.1 (U07611)
